# Cystatin C: A Primer for Pharmacists

**DOI:** 10.3390/pharmacy8010035

**Published:** 2020-03-09

**Authors:** Hilary R. Teaford, Jason N. Barreto, Kathryn J. Vollmer, Andrew D. Rule, Erin F. Barreto

**Affiliations:** 1 Department of Pharmacy, Mayo Clinic, Rochester, MN 55905, USA; teaford.hilary@mayo.edu (H.R.T.); barreto.jason@mayo.edu (J.N.B.); 2 College of Pharmacy and Health Sciences, Drake University, Des Moines, IA 50311, USA; Kathryn.vollmer@drake.edu; 3 Division of Nephrology and Hypertension, Mayo Clinic, Rochester, MN 55905, USA; rule.andrew@mayo.edu; 4 Division of Epidemiology, Mayo Clinic, Rochester, MN 55905, USA; 5 Robert D. and Patricia E. Kern Center for the Science of Health Care Delivery, Mayo Clinic, Rochester, MN 55905, USA

**Keywords:** cystatin C, biomarker, kidney function, pharmacokinetics, estimated glomerular filtration rate, muscle mass, sarcopenia, critical illness

## Abstract

Pharmacists are at the forefront of dosing and monitoring medications eliminated by or toxic to the kidney. To evaluate the effectiveness and safety of these medications, accurate measurement of kidney function is paramount. The mainstay of kidney assessment for drug dosing and monitoring is serum creatinine (SCr)-based estimation equations. Yet, SCr has known limitations including its insensitivity to underlying changes in kidney function and the numerous non-kidney factors that are incompletely accounted for in equations to estimate glomerular filtration rate (eGFR). Serum cystatin C (cysC) is a biomarker that can serve as an adjunct or alternative to SCr to evaluate kidney function for drug dosing. Pharmacists must be educated about the strengths and limitations of cysC prior to applying it to medication management. Not all patient populations have been studied and some evaluations demonstrated large variations in the relationship between cysC and GFR. Use of eGFR equations incorporating cysC should be reserved for drug management in scenarios with demonstrated outcomes, including to improve pharmacodynamic target attainment for antibiotics or reduce drug toxicity. This article provides an overview of cysC, discusses evidence around its use in medication dosing and in special populations, and describes practical considerations for application and implementation.

## 1. Introduction

Interpreting kidney function is central to dosing medications to achieve optimal effectiveness and safety. Approximately two-thirds of medications utilized are extensively eliminated by the kidney [1,2] and 23% of hospitalized patients are on one or more potentially nephrotoxic medications [3]. In both inpatient and ambulatory settings, the standard of care biomarker used to estimate kidney function for medication management is serum creatinine (SCr). Serum creatinine is an endogenous biomarker generated from skeletal muscle that undergoes passive clearance via glomerular filtration and active clearance through tubular secretion. SCr is ingrained in nearly all aspects of care including routine laboratory assessment (as with basic metabolic panels), clinical decision support, and drug information compendia [4,5,6]. Despite the frequency of SCr use and familiarity among clinicians, it has well-known limitations that make it a far from ideal marker of kidney function. SCr is influenced by numerous non-renal determinants that are incompletely accounted for in equations to estimate glomerular filtration rate (eGFR) including muscle mass, time of day, protein intake, pregnancy, hyperglycemia, drugs that interfere with tubular secretion, and volume status [7].

In the last decade, various biomarkers have emerged as adjuncts or alternatives to SCr. Generally, these tests fall into one of two categories, either a damage biomarker or a functional biomarker. Damage biomarkers such as neutrophil gelatinase-associated lipocalin (NGAL) may be helpful for early identification of acute kidney injury (AKI) before a rise in SCr is observable [8]. Functional biomarkers like SCr and serum cystatin C (cysC) characterize the capacity of the kidneys to filter solute [8]. CysC is a low-molecular-weight protein released from all nucleated cells [8]. It is freely filtered at the glomerulus prior to catabolism in the proximal tubule [8]. Serum cysC was incorporated into international guidelines for the identification of chronic kidney disease (CKD) in 2013 [9]. In a landmark trial in 2012, cysC in combination with SCr better predicted measured GFR (mGFR) than either biomarker used in isolation [10]. From these data, the Chronic Kidney Disease Epidemiology Collaboration (CKD-EPI) eGFR_creatinine_, eGFR_cystatinC,_ and eGFR_creatinine-cystatinC_ equations were developed (Supplementary Materials Table S1) [10]. In the same manner as SCr, the concentration of cysC is expected to be inversely correlated with GFR (Table 1) and the absolute concentration can be used in derived GFR-estimating equations (Supplementary Materials Table S1). Compared to SCr, cysC appears to be less affected by age, race, sex, muscle mass, or dietary intake [11]. However, cysC may still be influenced by some factors such as uncontrolled thyroid disease, corticosteroid use, smoking, elevated C-reactive protein (CRP) levels, coronary artery disease, and conditions with rapid cell turnover such as malignancies [4,12,13,14]. While serum cysC may have some features that may make it attractive for use in difficult to assess cases, it is important to underscore that all of these estimation equations, whether with SCr or cysC, perform poorly in rapidly changing renal function. The equations were developed in stable ambulatory care patients and depend on production and elimination of the biomarker being at steady state. In patients with dynamic kidney function (e.g., AKI, renal recovery), as is often the case in the critically ill, all estimation equations will lead to suspect results.

Pharmacists in both inpatient and ambulatory settings must have a nuanced appreciation for the selective use of emerging functional biomarkers like serum cysC given the potential impact that accurate estimation of kidney function has on medication management. This article provides an overview of cysC, discusses evidence and limitations for its use in medication dosing, challenges with its application in special populations, and practical considerations for implementation.

## 2. Overview of Measured Versus Estimated GFR

It is broadly agreed upon that mGFR derived from plasma or urinary disappearance of exogenous compounds such as inulin, iothalamate, ethylenediaminetetraacetic acid, or iohexol, is the reference standard for quantifying kidney function. Yet mGFR based on any one of these tools requires laboratory expertise with the testing method, the tests can be expensive, and they represent a single moment in time that limits bedside application in dynamic hospitalized patients. The frequency with which mGFR would need to be repeated for a dynamic inpatient evaluation renders this test generally less feasible than other approaches. Given these limitations of mGFR, more practical alternative approaches to estimate GFR from endogenous markers have been developed [7,15]. The historical standard was the Cockcroft-Gault (CG) estimated creatinine clearance (eCrCl) equation. This equation was developed in 1976 in a cohort of 249 Caucasian males, during which SCr was regressed along with other variables to predict measured urinary creatinine clearance [16]. The CG eCrCl equation predates creatinine assay standardization which occurred in the early 2000s. Several other equations to estimate kidney function have emerged since the development of the CG equation, including the Modification of Diet in Renal Disease (MDRD) and CKD-EPI equations [10,17]. These more contemporary eGFR equations were optimized for the prediction of mGFR [18,19]. This objective is distinct from developing an equation which predicts clinical outcomes such as future progression to end-stage kidney disease or mortality. Indeed SCr (and cysC), independent of mGFR, predict clinical outcomes [20]. This persistent association with outcomes despite accounting for underlying kidney function likely is explained by the non-renal determinants of each biomarker (e.g., muscle mass and inflammation, respectively) [21].

## 3. Medication Dosing Based on cysC

Just as eGFR equations were not optimized to predict clinical outcomes, these equations were also not initially developed or intended to predict drug clearance. Yet, they have been secondarily applied for this purpose as there is a need to have a clinically useful guide for adjusting drug doses. Antimicrobials, antithrombotics, cardiovascular medications, and chemotherapy are among the most commonly used renally-eliminated medications [3].

In the most recent 2010 draft guidance document for pharmacokinetic studies in patients with impaired kidney function, the Food and Drug Administration (FDA) acknowledged the infeasibility of routine use of mGFR in pharmacokinetic clinical trials to predict drug clearance [22]. The FDA indicated that the CG-eCrCl or the MDRD eGFR equation could preferentially be used to assign subjects to a categorical kidney impairment stage/threshold (e.g., ≥90 mL/min, 50–89 mL/min, 20–49 mL/min, <20 mL/min) for pharmacokinetic analyses of drugs. There was initial concern about the interoperability of these various kidney function equations for medication dose selection, but simulation data demonstrated good concordance between CG-eCrCl and MDRD eGFR for recommended drug doses [23].

While these simulated data are reassuring about the potential impact when changing between SCr-based formulae, introduction of an alternative biomarker into the equation, such as cysC, leads to considerably different results, particularly in acutely ill patients. In a study of 308 hospitalized adults treated for infections with both a SCr and a cysC checked, the authors simulated the impact that changing between the CKD-EPI eGFR_creatinine_, eGFR_cysC_, or eGFR_creatinine-cysC_ equations (expressed in mL/min) and the CG eCrCl would have on antibiotic dose selection [24]. The authors found that use of a cysC-only approach (eGFR_cysC_) or the use of both SCr and cysC in the eGFR_creatinine-cysC_ equation in this acutely ill population resulted in a substantial decrease in the agreement between selected doses to 49% and 68%, respectively. The majority of discordant doses would be lower if cysC-based equations were substituted for SCr-based equations [24]. As an example, in a critically ill patient with sepsis in the setting of reduced muscle mass where CG eCrCl is 90 mL/min, it is entirely feasible for the eGFR_creatinine-cysC_ and eGFR_cysC_ to be lower at approximately 50 and 30 mL/min, respectively. If applied to historical dosing thresholds based on eCrCl, use of either of these estimates could result in a decrease in antibiotic doses by as much as 50%. In acutely ill patients with high severities of illness and variable pharmacokinetic profiles, blanket application of new eGFR formulas with non-SCr biomarkers like cysC to historical renal dose recommendations derived from SCr-centric thresholds is ill advised. Rather, pharmacokinetic studies and new drug dosing algorithms should be developed and tailored to a specific kidney assessment tool.

The association between cysC and the corresponding eGFR and drug pharmacokinetics has been described in a number of studies [25]. A systematic review that explored the predictive relationship between cysC and drug clearance identified 28 studies published between 2004 and 2017 that included 3455 patients treated with 16 different renally-eliminated medications [25]. Antimicrobials were the most commonly studied and included vancomycin, aminoglycosides, beta-lactams, tenofovir, and teicoplanin. Other drugs evaluated included digoxin, dabigatran, perindopril, carboplatin, and topotecan. Overall, eGFR_cysC_ predicted observed drug clearance and serum concentrations of medications as well as, if not better than, SCr in the majority of the identified studies [25]. The literature was most robust for vancomycin, which was evaluated in 12 studies that included 1806 patients [26,27,28,29,30,31,32,33,34,35,36]. In six of these studies that reported vancomycin clearance (L/h), eGFR_cysC_ better predicted drug clearance than eGFR_creatinine_ (R^2^, eGFR_cysC_ = 0.70–0.85; eGFR_creatinine_ = 0.37) [25]. In the remaining six studies, trough levels were the outcome of interest and were better predicted by eGFR_cysC_ than eGFR_creatinine_ [pooled R^2^ (N = 191): eGFR_cysC_ = 0.54 (95% CI, 0.43–0.64) vs. eGFR_creatinine_ = 0.26 (95% CI 0.12–0.39)] [25]. Across all 28 included studies, equations for SCr- and cysC-based estimates of kidney function varied [25]. Since 2017, additional literature has been published that corroborated these findings [37,38,39]. These studies were primarily pharmacokinetic associations or simulations. They require testing and application in the clinical environment to evaluate the potential to improve pharmacodynamic target attainment and clinical outcomes.

One study applied empirical modeling to a cohort of 173 hospitalized patients to develop a prediction algorithm for initial steady state vancomycin troughs [30]. The optimal performing model, that included eGFR_creatinine-cysC_, weight, vancomycin dose, and dosing interval, was projected to increase vancomycin target achievement 2.5-fold compared to the same model with eGFR_creatinine_ [30]. This model was translated into a simple dosing algorithm and implemented in an independent validation cohort. When compared to standard dosing in a follow-up study, the authors found significantly reduced inter-individual drug level variability (P < 0.001) and a 2-fold increase in target trough achievement compared to standard care (28% to 50%; P < 0.001) [29]. While promising, future studies need to explore these proof-of-principle findings in patients most likely to benefit from vancomycin optimization, including in those with microbiologically-confirmed resistant Gram-positive infections in which the drug is essential. This framework could be extended to other renally-eliminated medications where dose optimization is needed.

## 4. Special Populations with cysC

**Figure 1:**
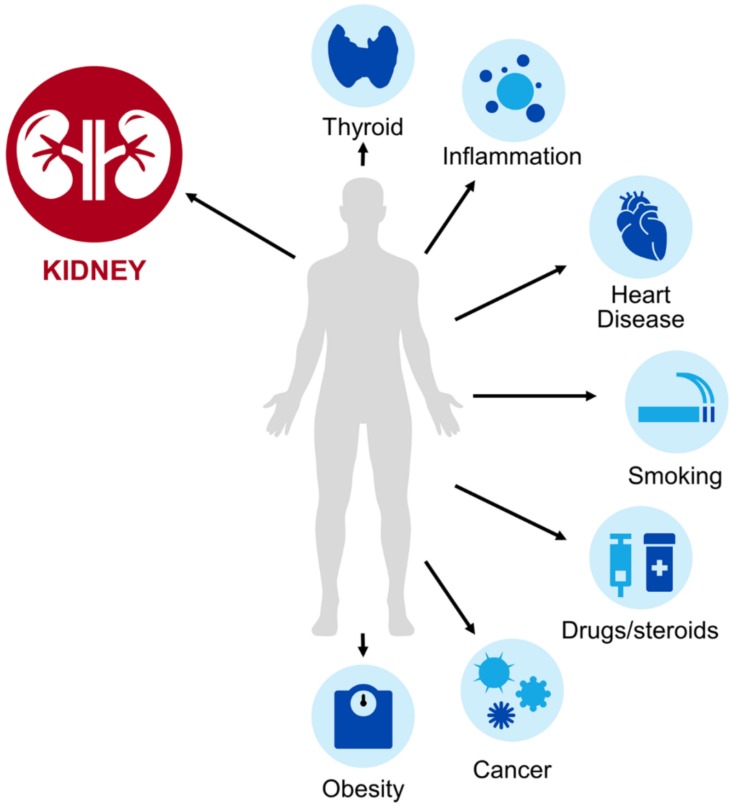
Select determinants of serum cystatin C (cysC) levels.

As cysC is a proteinase inhibitor found in all nucleated cells and involved in the inflammatory cascade, it has multiple physiologic functions and potential non-renal factors that influence its interpretation (Figure 1) [8]. To draw a parallel, pharmacists could likely recognize that patients with low muscle mass have low SCr production which could lead to an overestimation of kidney function. Similarly, pharmacists should be aware of populations where cysC-based estimates of kidney function may be uniquely helpful or uniquely problematic (Table 2). These factors are especially important in patient subsets not well characterized in the outpatient eGFR equation validation studies [10]. Unlike with SCr, where pharmacokinetic studies have focused on special populations with a suspected high prevalence of non-renal determinants (i.e., elderly), these are rarely included in the existing cysC pharmacokinetic studies. Until such information is available in the context of a pharmacokinetic evaluation, it is reasonable to apply insights gained from literature focused on GFR or biomarker concentrations in general to understand factors pharmacists should consider when interpreting cysC values. This section will focus on key studies regarding patients with obesity, altered muscle mass, cancer, treated with corticosteroids, or with a solid organ transplant. In our experience, these are particularly relevant scenarios to consider when cysC is applied to acute care medication management. While not elaborated on in detail in this review, other factors such as smoking [13], cardiovascular disease [11], thyroid disorders [12,40,41], diabetes [11], and inflammation [11] have also been linked to increased cysC and should be considered in its interpretation. Several of these factors may coexist in a single patient.

Cystatin C is primarily eliminated renally via glomerular filtration, but as an endogenous biomarker, its concentration can be affected by other non-renal determinants (depicted in the light blue circles in Figure 1). These factors may lead to altered cysC production, which can change observed serum concentrations and corresponding GFR estimates. Clinicians must be aware of these factors to accurately interpret cysC for kidney assessment. Additional detail on the use of cysC in these special populations is provided in Section 4 and Table 2.

### 4.1. Obesity

Kidney function assessment in obese patients is challenging, with or without the use of cysC. Kidney size, kidney function, and body size are proportionally related to a point; however, the change becomes nonlinear at the extremes of weight [42]. In obese patients, a 2-fold increase in body weight results in only a 1.6-fold higher mean GFR [42]. As weight increases, nephron number does not change. Rather, the increase in GFR observed in obese individuals reflects compensatory hyperfiltration in the nephrons that do exist. This hyperfiltration in obese patients can become maladaptive and is largely unaccounted for in existing eGFR equations [43].

Several issues exist with the use of cysC to interpret kidney function in obese patients. Obese individuals have elevated cysC levels, independent of underlying GFR as adipose tissue has a uniquely high expression of cysC compared to other cell types [11]. This means that independent of underlying kidney function, the biomarker may be elevated and consequently lead to an underestimated GFR. Another aspect of using eGFR for drug dosing in obesity is whether or not to index the eGFR to body surface area (BSA) [43]. In the validated CKD-EPI eGFR_cysC_ or eGFR_creatinine-cysC_ equations, kidney function is expressed in milliliters per minute per BSA (mL/min/1.73 m^2^) [44]. Historically, a BSA of 1.73 m^2^ was the mean value for an average 25-year-old American male, but recent data suggest that it is closer to 2.05 m^2^ [43,45]. Studies have questioned the appropriateness of the BSA correction value of 1.73 m^2^ and which BSA estimation strategy should be used [43,44,46,47,48,49]. Take the example of a 126.7 kilogram patient, who has a calculated BSA between 2.41 and 2.56 m^2^, depending on the BSA equation used [43]. The eGFR for this obese patient might be approximately 70 mL/min, but if an indexed eGFR value were used instead, the value would be 47–50 mL/min/1.73 m^2^. The different units of measure may go unrecognized in practice or may be variably interpreted by bedside clinicians. When possible, in both obese and non-obese patients, it is advisable to dose medications based on kidney function expressed in mL/min. In this example with the 126.7 kg patient, use of the eGFR expressed in mL/min/1.73 m^2^ would have led to a dose decrease for many renally-eliminated medications, which could correspond to decreased effectiveness or undertreatment. Obesity is a challenging issue when interpreting cysC. Clinicians should be aware of the increased cysC production in obesity independent of GFR, the local eGFR_cysC_ or eGFR_creatinine-cysC_ equations used, and their corresponding units of measure. If using eGFR_cysC_ or eGFR_creatinine-cysC_ for drug dosing, clinicians should use the equation = eGFR in mL/min/1.73 m^2^ × [BSA (m^2^)/1.73 m^2^] to express in mL/min.

### 4.2. Disorders of Compromised Muscle Mass or Cachexia

As the terminal byproduct of skeletal muscle catabolism, in patients with altered muscle mass, the observed SCr may be more influenced by its production than its elimination. Consequently, patients may have a low SCr not because of increased renal elimination, but rather because of reduced production [50]. Their relatively high corresponding SCr-based eGFR in this scenario is likely an overestimation of true kidney function. CysC concentration is independent of skeletal muscle, thus it can be a highly useful tool to estimate kidney function in patient subgroups with reduced muscle mass [51,52,53].

The impact of various disorders on the accuracy of eGFR estimations using cysC has been a topic of interest. In a comparison of amyotrophic lateral sclerosis (ALS) patients and healthy controls with a similar mean eGFR_cysC,_ the ALS group had a 283 mL/min/1.73 m^2^ higher mean eGFR_creatinine_ [52]. In a study of 20 hospitalized children and adolescents with Duchenne muscular dystrophy, bias of different eGFR equations for mGFR was assessed. In this study and throughout the remainder of this review, bias is defined as the difference between mGFR and eGFR (mGFR-eGFR). In this cohort, eGFR_creatinine_ equations overestimated mGFR by up to 300%, with a bias of −327.7 mL/min/1.73 m^2^ for the SCr-based Schwartz equation (Supplementary Materials Table S1) [53]. Estimated GFR_cysC_ using the Filler equation (Supplementary Materials Table S1) best approximated mGFR in this cohort with a bias of 10.9 mL/min/1.83 m^2^ [53]. Improved accuracy of GFR estimates using cysC compared to SCr has also been observed in spinal cord injury and cystic fibrosis patients [54,55]. In amputees, eGFR_cysC_ remains unaffected following amputation, whereas an otherwise normal eGFR_creatinine_ decreases by approximately 20 mL/min/1.73 m^2^ [56]. In general, use of cysC is likely to produce more accurate estimates of kidney function compared to SCr in those with reduced muscle mass due to disease or amputation.

Patients with long hospitalizations or those with hypercatabolic states are also subject to muscle wasting. In 158 critically ill patients without evidence of AKI at admission or during their stay, over 7 days, the SCr decreased by 25% on average. During that same interval, no change in cysC was observed [57]. Hospital length of stay is expected to be proportionally related to the degree of discrepancy_._ Given that the accuracy of kidney function assessment can have critical implications for drug dosing, fluid management, diagnostic testing, and transitions to higher or lower levels of care, cysC testing may be a helpful test in those with deconditioning or loss of muscle in the hospital.

While ample evidence explores the bias and precision of eGFR for mGFR in patients with compromised muscle mass, fewer studies have focused on how these differences translate to medication use. One study of 38 amputees compared the subjects’ prescribed medication doses to those recommended from Renbase^®^, a clinical decision support software for drug dose adjustments_._ When applying eGFR_creatinine_, eGFR_creatinine-cysC_ and eGFR_cysC_ against the Renbase^®^ thresholds, 10.8%, 17.6% and 37.8% of patients, respectively, were found to have at least one medication prescribed differently than recommended [58]. In a population-based pharmacokinetic study of vancomycin use in 37 spinal cord injury patients, the CKD-EPI eGFR_cysC_ equation better predicted steady-state troughs than other eGFR equations [28]. Similar findings have been observed in hospitalized elderly patients, another population likely to have reduced muscle mass [59]. More studies are needed of pharmacokinetic and clinical outcomes with cysC-guided dosing protocols in populations with reduced muscle mass. Evidence does not support directly substituting eGFR_cysC_ into SCr-based cutoffs, but in cases where the SCr is clearly inaccurate, this approach may be necessary. Measured GFR, therapeutic drug monitoring with drug levels, and close monitoring of toxicity and effectiveness should supplement any use of cysC-based eGFR equations to guide drug management in these scenarios.

### 4.3. Malignancy

CysC has been evaluated in patients with hematologic and oncologic malignancies for both its renal and its non-renal properties. While cysC is produced by all nucleated cells and secreted into the blood at a constant rate, cancer and its related treatments can cause highly variable and often heightened degrees of cysC release, which makes its use in this population challenging [59,60,61].

Some studies demonstrate a baseline cysC level that is approximately 0.3mg/L higher in cancer patients than in healthy controls with comparable kidney function [62,63,64]. The impact of a 0.3 mg/L cysC increase has varying effects on eGFR depending on age, sex, and baseline cysC (Table 1). Several studies suggest an inverse relationship between cysC and cancer prognosis. In colorectal cancer, lung cancer, melanoma, and diffuse large B-cell lymphoma, an elevated cysC level has been associated with worse cancer outcomes [62,63,65,66]. In these cancers, it is primarily the non-renal aspects of cysC that are being exploited for prognostic purposes. A disease state in which both the renal and non-renal aspects of cysC are significant is multiple myeloma, a cancer commonly associated with kidney dysfunction. In a cohort of symptomatic myeloma patients, cysC-based eGFR more frequently led to a CKD stage 3–5 diagnosis compared to the SCr-based MDRD eGFR equation [64]. CysC also has a strong correlation with myeloma tumor burden [67]. Kidney dysfunction is a commonly known complication of multiple myeloma with as many as 40% of patients having some amount of kidney impairment at the time of their multiple myeloma diagnosis [68]. The complex dynamic between the direct effects of multiple myeloma on the kidney (due to light or heavy chain deposition) and indirect effects (when myeloma in the bone increases calcium secretion leading to calcium deposits in the kidney) have yet to be fully understood. However, any insult to the kidney causes a release of cysC that is directly proportional to the severity of multiple myeloma, making cysC a viable surrogate marker of disease and treatment response [67]. Longitudinal trends in serum cysC concentrations strongly predicted outcomes in myeloma including overall survival [64,67,69]. Given that survival in multiple myeloma is inversely related to the diagnosis and severity of CKD, use of cysC to more accurately diagnose CKD and prognosticate could inform risk/benefit discussions about the use of aggressive chemotherapy [64].

Outside of cancer-related outcomes, researchers have investigated the utility of cysC as a tool to renally-dose chemotherapy. The narrow therapeutic indices of chemotherapeutics are one area where accurate kidney assessment is essential for medication safety and effectiveness. Studies with platinum-agents, particularly carboplatin, which are renally-eliminated and nephrotoxic, demonstrated that cysC predicted carboplatin area under the curve and clearance at least as well as SCr (R^2^ for drug clearance 0.71 with SCr and 0.8 with cysC) [61,70,71,72]. Use of cysC to more accurately predict drug clearance could spare patients dose-dependent adverse drug reactions such as carboplatin-induced thrombocytopenia [73]. Another example of when cysC may be beneficial is with dosing and monitoring high-dose methotrexate, which is known to have substantial systemic toxicity including effects on the kidney [74]. CysC has also been shown to be superior to SCr for prediction of topotecan elimination, which could reduce myelosuppression and neutropenia-related complications without compromising treatment effectiveness [75]. While only limitedly studied, use of both SCr and cysC in combination to assess kidney function may be a future direction for research in cancer patients. Preliminary data suggest that this approach may facilitate more appropriate drug dosing, better detect GFR changes, and, optimize cancer care [76,77].

The true role of cysC for cancer prognostication and GFR-assessment in patients with cancer remains undefined. Clinicians should review literature surrounding cysC utilization specific to the cancer subtype and the drug of interest prior to widespread adoption into everyday clinical practice.

### 4.4. Corticosteroids

CysC concentration may be elevated in the presence of corticosteroids independent of underlying kidney function. Proposed mechanisms include a promoter-mediated increase in cysC transcription or increased activity at the glucocorticoid receptor, though the exact mechanism remains poorly understood [78,79]. The impact of corticosteroids on cysC concentrations has been examined in various populations with conflicting results. Studies in graves ophthalmopathy [80], asthma [81], symptomatic heart failure [82], and solid tumors [60] have noted increases in cysC independent of GFR in adults receiving short courses of relatively high doses of corticosteroids (>30 mg prednisone equivalent for <2 weeks). The typical magnitude of these increases is 0.2–0.4 mg/L, or a 25% reduction in eGFR for most patients. CysC usually peaks 2–3 days after steroid administration and the effect diminishes by 7 days. Findings in children have been more conflicting, with some studies finding no or limited changes in cysC after administration of high-dose short-course steroids or during chronic steroid administration, while others found dose-dependent increases in cysC [83,84,85,86].

In studies which included mGFR, corticosteroids exhibited dose-dependent effects on the inaccuracy of eGFR_cysC._ In 50 hospitalized rheumatoid arthritis patients receiving ≥10 mg/day of prednisone, eGFR_cysC_ using the Japanese Society of Nephrology (JSN) equation (Supplementary Materials Table S1) underestimated inulin clearance by 15.3 mL/min/1.73 m^2^ (49.8 ± 23.0 mL/min/1.73 m^2^ vs. 65.1 ± 30.5 mL/min/1.73 m^2^) [87]. The authors found that the average of the JSN eGFR_creatinine_ and JSN eGFR_cysC_ was the most accurate for prediction of mGFR in those receiving ≥10 mg of prednisone daily. Chronic prednisone doses of <10 mg daily did not alter the accuracy of the JSN eGFR_cysC_, which was the most accurate equation in the low-dose steroid group for prediction of mGFR among the three equations evaluated (JSN eGFR_creatininine_, JSN eGFR_average_ and JSN eGFR_cysC_)(Supplementary Materials Table S1) [87].

The impact of corticosteroids on medication dosing remains unclear. Corticosteroid use is seldom incorporated as a factor in dosing models with cysC. In a study that explored determinants of gentamicin clearance, the CKD-EPI eGFR_cysC_ and eGFR_creatinine-cysC_ equations similarly predicted drug clearance in the 41/260 patients in the cohort who received corticosteroids or had abnormal thyroid function tests compared to those who did not [88]. However, this was a small sample size, steroid doses were not reported, and the steroid recipients were studied in combination with the thyroid dysfunction subgroup. Given the high prevalence of corticosteroid use in hospitalized patients, drug dosing studies should examine the relative performance of models with and without this factor.

### 4.5. Transplant

Prior to transplantation, solid organ transplant candidates typically experience chronic illness, immobility, and poor nutrition. These factors may lead to altered, and often decreased SCr production independent of kidney function due to muscle wasting [89]. Chronic inflammation can lead to slight increases in baseline cysC levels. After transplantation, the pro-inflammatory state and introduction of immunomodulatory therapies can elevate cysC independent of GFR [90]. This potential post-transplant elevation in cysC level was demonstrated in a study comparing 206 transplant recipients and 204 chronic kidney disease patients. At the same cysC level, the transplant recipients had a 19% higher true GFR than the non-transplant patients with chronic kidney disease [90].

The use of cysC has commonly been examined in transplant recipients for the prediction of GFR or clinical outcomes such as need for dialysis or mortality. While the next two sections will detail this evidence for various transplant types and time points throughout transplant care, one must recognize that prediction of GFR, graft survival, dialysis, and mortality does not equate to prediction of medication clearance. Instead, pharmacokinetic models specific to the transplant population should be developed to better examine prediction of medication clearance in this subgroup.

#### 4.5.1. Kidney Transplant

Trends in cysC immediately after kidney transplant or during acute rejection may not correspond predictably with those of SCr or mGFR. CysC has been shown to decline immediately post-transplant, even in those with delayed graft function, a finding attributed to rapid metabolism of cysC by tubular cells immediately after reperfusion of the new organ [91]. CysC has also been reported to paradoxically increase in the week following kidney transplant despite improving mGFR and adequate graft function, typically attributed to high-dose methylprednisolone (≥500 mg) in the operative setting [91,92]. CysC also appears elevated independent of GFR in the setting of acute rejection likely due to the treatment with high-dose steroids [91]. Clinicians should thus consider cysC unreliable in the first week following kidney transplant and during treatment episodes of acute rejection.

CysC has been extensively studied in the stable post-kidney transplant population. CysC predicts graft survival. However, its superiority over SCr has not been consistently established for this purpose [93,94]. For GFR estimation using the reference standard of mGFR, authors have applied equations (Supplementary Materials Table S1) that were derived from both non-transplant patients (CKD-EPI, MDRD) and transplant recipients (Rule [90] or Le Bricon [95] equation). Results have been mixed. Compared to SCr-based equations, some have found no improvement in prediction of mGFR or CKD classification with CKD-EPI eGFR_creatinine-cysC_ [96,97]. Others have found reduced bias for mGFR or measured creatinine clearance with the Le Bricon [98,99], Filler [100,101], or combined equations such as the JSN eGFR_average_ or the CKD-EPI eGFR_creatinine-cysC_ equations [102]. With evolution of new equations over time and changes in cysC assays over the years, a clear “best” equation in stable kidney transplant recipients is difficult to identify. Importantly, the bias observed for mGFR using various eGFR equations with SCr, CysC, or both is commonly less than 10 mL/min/1.73 m^2^ [98]. Other populations that we have already touched on, such as patients with cachexia and neuromuscular disorders, are expected to have much more discrepant eGFR values. This can lead to more significant medication-dosing dilemmas. In absence of an established optimal equation for stable post-renal transplant patients, pharmacists should apply similar principles to kidney function assessment as in other populations.

#### 4.5.2. Non-Kidney Transplant

CysC use in patients with cirrhosis and liver transplant recipients has garnered attention given the regularity with which this population exhibits poor nutrition, reduced muscle mass, and chronic critical illness. Numerous studies have demonstrated improved accuracy and precision of eGFR_cysC_ and eGFR_creatinine-cysC_ for mGFR in those with cirrhosis compared to eGFR_creatinine_ [103,104]. One study demonstrated that the concentration of cysC alone may predict mortality more accurately than the MELD score, which is likely a reflection of both its renal and non-renal properties [105]. Pre-transplant cysC has also shown to independently predict post-transplant mortality [105,106,107,108]. In cirrhotic patients, cysC can be considered as an adjunct or alternative for kidney function assessment. Although given the level of evidence to date, it would be premature to apply these data to organ allocation.

Limited evidence for cysC use exists in heart and lung transplant. The immediate post-transplant period has been studied in heart transplant patients. In 117 heart transplant patients, those with AKI had a significantly higher cysC level compared to those without AKI just 3 h after surgery [109]. In contrast, SCr was similar between those with AKI and those without until 4 days after surgery. In the same study, a persistently elevated (≥2.54 mg/L) cysC at 7 days was an independent predictor of 1 year mortality [109]. The stable post-transplant period has been examined in lung transplant recipients, which, unsurprisingly, found that SCr-only equations were inadequate in those with low muscle mass, BMI <20 kg/m^2^, or in those on trimethoprim-containing antibiotics [110]. Despite some early evidence, more is needed prior to broad application of cysC in heart and lung transplant recipients.

## 5. Implementation

CysC is one of the many new kidney function assessment tools available to pharmacists after decades of a SCr-only approach [8]. Health care settings without access to cysC are limited in their ability to rapidly assess kidney function in these special populations where SCr values are highly discrepant for mGFR prediction. Barriers to cysC testing in the acute care setting have not been fully described but turnaround time seems to be one. Another determinant of cysC use may be the cost of testing. While the reagents for cysC are more expensive than for SCr ($4 per test for cysC versus $0.20 for SCr), cysC can be run on the same laboratory technology as SCr and the test is less expensive than many other commonly performed tests including Troponin T and B-type natriuretic peptide [4]. The approach to result reporting may also influence use. While the absolute cysC concentration is unlikely to carry much significance for non-nephrologists, coupling it with eGFR is likely to enhance usability of the results. Sites should work with laboratory leadership to report eGFR_cysC_ in addition to absolute cysC concentration to maximize utility of the tool.

**Figure 2:**
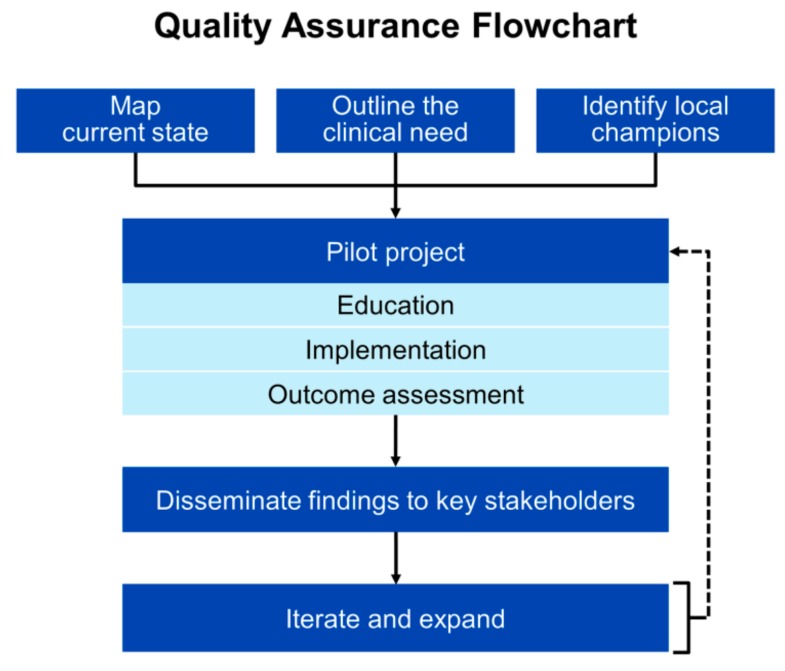
Example of an implementation roadmap to accelerate translation of novel renal biomarkers to the bedside.

Extrapolating evidence from implementing other new laboratory tests leads to the conclusion that applying the principles of quality improvement could be helpful to accelerate translation of novel renal biomarkers to the bedside. Steps include engaging local champions, designing a pilot, educating clinicians, developing and piloting the workflow, monitoring for protocol adherence and problems, collecting outcomes, modifying the protocol as needed, and disseminating the findings [111] (Figure 2). Relating this model to our experience with cysC, a vancomycin dosing quality improvement project served as the pilot project that allowed for systematic introduction of cysC to clinicians [8]. This initiative, coupled with the release of the 2012 CKD-EPI equations, likely heightened clinician familiarity with the test to the point where they naturally sought more broad application.

**Figure 3:**
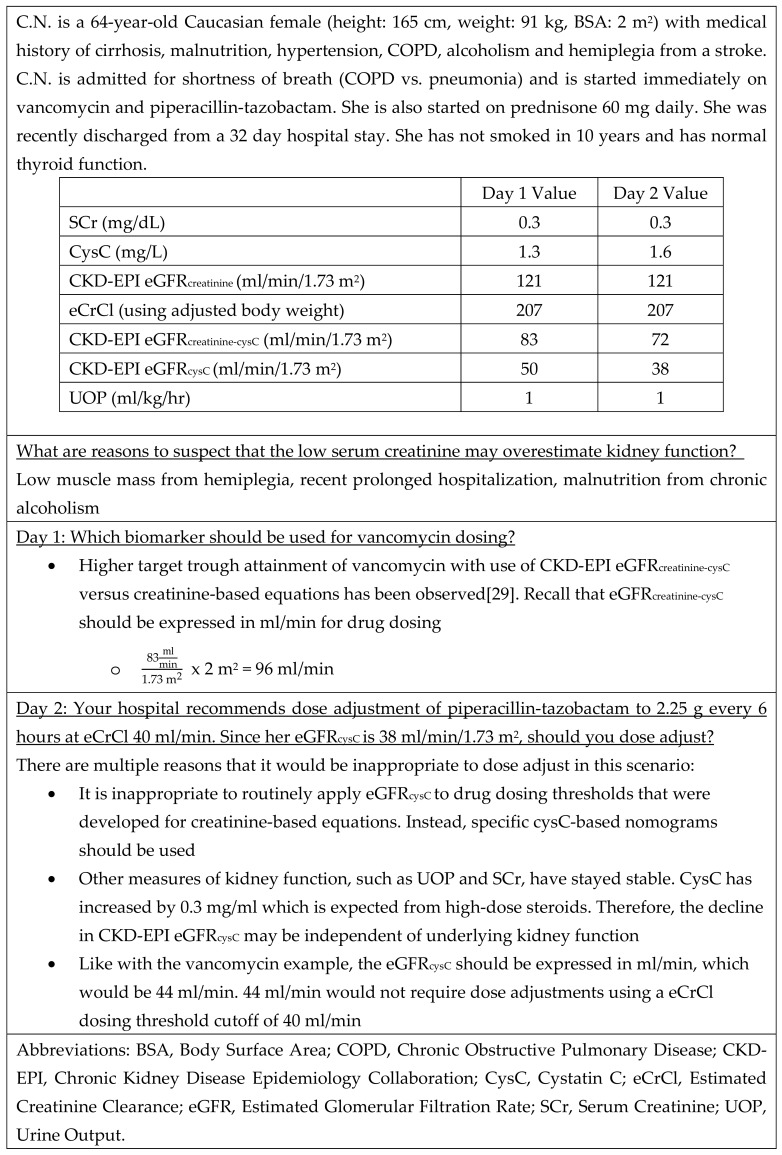
Patient Case Example.

Clinicians also need education about the various non-renal confounders of cysC concentration and considerations with applying eGFR_cysC_ to medication dosing and monitoring. Institutions that are looking to further incorporate cysC into their practice should provide structured education around use and guidance for testing in specific patient populations with sufficient evidence to indicate benefit (i.e., vancomycin patients, patients with altered muscle mass). Key elements of educational bundles could include (1) limitations of existing biomarkers, (2) potential indications for cysC testing, (3) non-renal confounders, and (4) logistical issues (e.g., test turnaround time, eGFR equations). Case vignettes such as that presented in Figure 3 could be used. 

Besides cysC, other examples of emergent kidney assessment tools include β-trace protein, β2 microglobulin, NGAL, TIMP2-IGFB7, and fluorescent injectates for “real-time” GFR assessment [4]. Guidance and best practice around these tools, specifically in the inpatient setting, remain limited. Efforts are needed to ensure rationale use of these tests and avoid laboratory test overuse, which is often present in low-volume tests (<100,000 tests per year) [112]. Explicit education and strategic implementation of these tools are necessary to avoid harm, waste, or confusion. As use of cysC continues to grow, lessons learned with this relatively inexpensive accessible test can be applied to facilitate seamless translation of other novel approaches to kidney assessment into practice.

## 6. Conclusions

After decades of a SCr-only approach, cysC has emerged as a new tool to improve medication use. CysC is produced independent of muscle mass, which makes it particularly appealing in populations with cachexia, prolonged hospitalizations, spinal cord injury, muscular dystrophy, amputations, and cirrhosis. Compared to SCr, cysC has also shown improved prediction of clearance for many medications, most notably vancomycin. Despite promising use cases, non-renal determinants of cysC must be considered including corticosteroid therapy, malignancy, cardiovascular disease, smoking, abnormal thyroid function, and obesity.

Pharmacists are key stakeholders in kidney function assessment and can play an integral role in rational incorporation of cysC into practice to improve medication use across the care spectrum. This work may include research on the applied use of cysC testing to improving medication effectiveness and safety, education of care teams, or implementation of clinical decision support. Pharmacists can capitalize upon their medication expertise and prominent role on the care team to deliver innovative medication initiatives utilizing cysC to the bedside.

## Figures and Tables

**Table 1:** Relationship between various cystatin C concentrations and estimated glomerular filtration rate.

	Serum Cystatin C Concentration (mg/L)				
Description	0.6	0.9	1.2	1.5	1.8
eGFR_cystatinC_ of a 50-year-old white male	126	93	64	47	37
eGFR_cystatinC_ of a 80-year-old black female	104	77	53	39	31
All eGFR expressed in mL/min/1.73 m^2^ and calculated from the CKD-EPI eGFR_cystatinC_ equation					

Abbreviations: CKD-EPI, Chronic Kidney Disease Epidemiology Collaboration; eGFR, Estimated Glomerular Filtration Rate.

**Table 2:** Key considerations with cysC use in special populations.

Population	Effect on Estimation	Considerations
Obesity [11]	eGFR_cysC_ could underestimate mGFR	CysC elevated independent of GFR compared to non-obese eGFR used for drug dosing should be expressed in mL/min (not mL/min/1.73 m^2^)
Compromised muscle mass or cachexia [51,52,53,54,55,56,57,58]	eGFR_cysC_ could more accurately predict mGFR	Favorable evidence around vancomycin dosing in spinal cord injuries and in the elderly
Malignancy [60,61,62,63,64,65]	eGFR_cysC_ could underestimate mGFR	0.1–0.3 mg/L cysC increase in presence of malignancy (varies by cancer type, independent of GFR) Potential benefit in predicting clearance of some chemotherapeutics including carboplatin and topotecan
Corticosteroids [79,80,81,82,83,84,85,86,87,88]	eGFR_cysC_ could underestimate mGFR	0.2–0.4 mg/L increase in presence of high-dose steroids (independent of GFR) Increases at a peak of 2–3 days after steroid administration, with the concentration returning to baseline within 7 days of administration Minimal impact with prednisone doses < 10 mg Pediatric studies on the impact of steroids on cysC values have been conflicting
Solid-organ transplant recipients [92,93,94,95,96,97,98,99,100,101,102,103,104,105,106,107,108,109,110,111]	eGFR_cysC_ could underestimate mGFR	CysC elevated independent of GFR compared to non-transplant patients (~20% elevation) Unpredictable in first week following kidney transplant or during treatment for acute rejection Best eGFR equation to use remains unclear
Cardiovascular disease [11]	eGFR_cysC_ could underestimate mGFR	CysC elevated independent of GFR
Smoking [13]	eGFR_cysC_ could underestimate mGFR	CysC elevated independent of GFR
Thyroid disorder [12,40,41]	Hypothyroid eGFR_cysC_ could overestimate mGFRHyperthyroid eGFR_cysC_ could underestimate mGFR	CysC levels lowered in uncontrolled hypothyroidism (0.1–0.4 mg/L decrease), independent of GFR CysC elevated in uncontrolled hyperthyroidism (0.1–0.4 mg/L increase), independent of GFR

Abbreviations: CysC, Cystatin C; GFR, Estimated Glomerular Filtration Rate; mGFR, Measured Glomerular Filtration Rate.

## Supplementary Materials

The following are available online at https://www.mdpi.com/2226-4787/8/1/35/s1, Table S1: Select kidney function estimation equations.
