# Supplementary material for: Cystatin C: A Primer for Pharmacists
Source: Pharmacy (Basel). 2020 Mar 9;8(1):35. doi: 10.3390/pharmacy8010035 (PMC7151673; doi:10.3390/pharmacy8010035)
Supplement: Supplementary file 1 [file pharmacy-08-00035-s001.pdf]

Supplemental Table 1: Select Kidney Function Estimation Equations

**Serum Creatinine-Based Equations**[a]	
CKD-EPI $eGFR_{creatinine}$[1]	$eGFR = 141 \times \min(SCr/\kappa,1)^{\alpha} \times \max(SCr/\kappa,1)^{-1.209} \times 0.993^{age} \times 1.018$ [if female] $\times 1.159$ [if black]<br>$\kappa = 0.7$ (females) or 0.9 (males)<br>$\alpha = -0.329$ (females) or -0.411 (males)
Cockcroft-Gault[2]	$eCrCl^{b} = \frac{(140 - age) \times (\text{weight in kg}) \times 0.85 \text{ [if female]}}{(72 \times SCr)}$
Japanese Society of Nephrology $eGFR_{creatinine}$[3]	$eGFR = 194 \times SCr^{-1.094} \times age^{-0.287} \times 0.739$ [if female]
MDRD[4]	$eGFR = 175 \times SCr^{-1.154} \times age^{-0.203} \times 0.742$ [if female] $\times 1.212$ [if black]
Schwartz[5]	$eGFR = \frac{0.413 \times (\text{height in cm})}{SCr}$
**Cystatin C-Based Equations**[a]	
CKD-EPI $eGFR_{cystatinC}$[1]	$eGFR = 133 \times \min(cysC/0.8, 1)^{-0.499} \times \max(cysC/0.8, 1)^{-1.328} \times 0.996^{age} \times 0.932$ [if female]
Rule[6]	$eGFR = 76.6 \times cysC^{-1.16}$
Filler[7]	$eGFR = 91.62 \times (1/cysC)^{1.123}$
Japanese Society of Nephrology $eGFR_{cystatinC}$[8]	$eGFR = 96 \times cysC^{-1.324} \times 0.996^{age} \times 0.894$ [if female]
Le Bricon[9]	$eGFR = [(78 \times (1/cysC)] + 4$
**Combined Equations**[a]	
CKD-EPI $eGFR_{creatinine\text{-}cystatinC}$[1]	$eGFR = 135 \times \min(SCr/\kappa, 1)^{\alpha} \times \max(SCr/\kappa, 1)^{-0.601} \times \min(SCr/0.8, 1)^{-0.375} \times \max(SCr/0.8, 1)^{-0.711} \times 0.995^{Age} \times 0.969$ [if female] $\times 1.08$ [if black]<br><br>$\kappa = 0.7$ (females) or 0.9 (males)<br>$\alpha = -0.248$ (females) or -0.207 (males)
Japanese Society of Nephrology $eGFR_{average}$[8]	Average of Japanese Society of Nephrology $eGFR_{creatinine}$ and Japanese Society of Nephrology $eGFR_{cystatinC}$
[a]: All eGFR expressed in mL/min/1.73 m^2<br>[b]: Creatinine clearance expressed in mL/min	
Abbreviations- CKD-EPI; Chronic Kidney Disease Epidemiology Collaboration, CysC; Cystatin C, eGFR; Estimated Glomerular Filtration Rate, eCrCl: Estimated Creatinine Clearance, MDRD; Modification of Diet in Renal Disease, SCr; Serum Creatinine	

References

1. Inker, L.A.; Schmid, C.H.; Tighiouart, H.; Eckfeldt, J.H.; Feldman, H.I.; Greene, T.; Kusek, J.W.; Manzi, J.; Van Lente, F.; Zhang, Y.L.; et al. Estimating glomerular filtration rate from serum creatinine and cystatin C. *N. Engl. J. Med.* **2012**, *367*, 20–9.
2. Cockcroft, D.W.; Gault, M.H. Prediction of creatinine clearance from serum creatinine. *Nephron* **1976**, *16*, 31–41.
3. Matsuo, S.; Imai, E.; Horio, M.; Yasuda, Y.; Tomita, K.; Nitta, K.; Yamagata, K.; Tomino, Y.; Yokoyama, H.; Hishida, A.; et al. Revised Equations for Estimated GFR From Serum Creatinine in Japan. *Am. J. Kidney Dis.* **2009**, *53*, 982–992.
4. The Modification of Diet in Renal Disease Study: Design, Methods, and Results From the Feasibility Study. *Am. J. Kidney Dis.* **1992**, *20*, 18–33.
5. Schwartz, G.J.; Muñoz, A.; Schneider, M.F.; Mak, R.H.; Kaskel, F.; Warady, B.A.; Furth, S.L. New equations to estimate GFR in children with CKD. *J. Am. Soc. Nephrol.* **2009**, *20*, 629–637.
6. Rule, A.D.; Bergstralh, E.J.; Slezak, J.M.; Bergert, J.; Larson, T.S. Glomerular filtration rate estimated by cystatin C among different clinical presentations. *Kidney Int.* **2006**, *69*, 399–405.
7. Filler, G.; Lepage, N. Should the Schwartz formula for estimation of GFR be replaced by cystatin C formula? *Pediatr. Nephrol.* **2003**, *18*, 981–985.
8. Horio, M.; Imai, E.; Yasuda, Y.; Watanabe, T.; Matsuo, S. GFR estimation using standardized serum cystatin C in Japan. *Am. J. Kidney Dis.* **2013**, *61*, 197–203.
9. Le Bricon, T.; Thervet, E.; Froissart, M.; Benlakehal, M.; Bousquet, B.; Legendre, C.; Erlich, D. Plasma cystatin C is superior to 24-h creatinine clearance and plasma creatinine for estimation of glomerular filtration rate 3 months after kidney transplantation [1]. *Clin. Chem.* **2000**, *46*, 1206–1207.
